# Correction: Expanded scope of Griesbaum co-ozonolysis for the preparation of structurally diverse sensors of ferrous iron

**DOI:** 10.1039/d1ra90167b

**Published:** 2021-11-19

**Authors:** Jun Chen, Ryan L. Gonciarz, Adam R. Renslo

**Affiliations:** Department of Pharmaceutical Chemistry, University of California San Francisco California 94143 USA adam.renslo@ucsf.edu

## Abstract

Correction for ‘Expanded scope of Griesbaum co-ozonolysis for the preparation of structurally diverse sensors of ferrous iron’ by Jun Chen *et al.*, *RSC Adv.*, 2021, **11**, 34338–34342, DOI: 10.1039/d1ra05932g.

The authors regret that an incorrect version of Fig. S1 was included in the original article. The correct version of Fig. S1 is presented below.
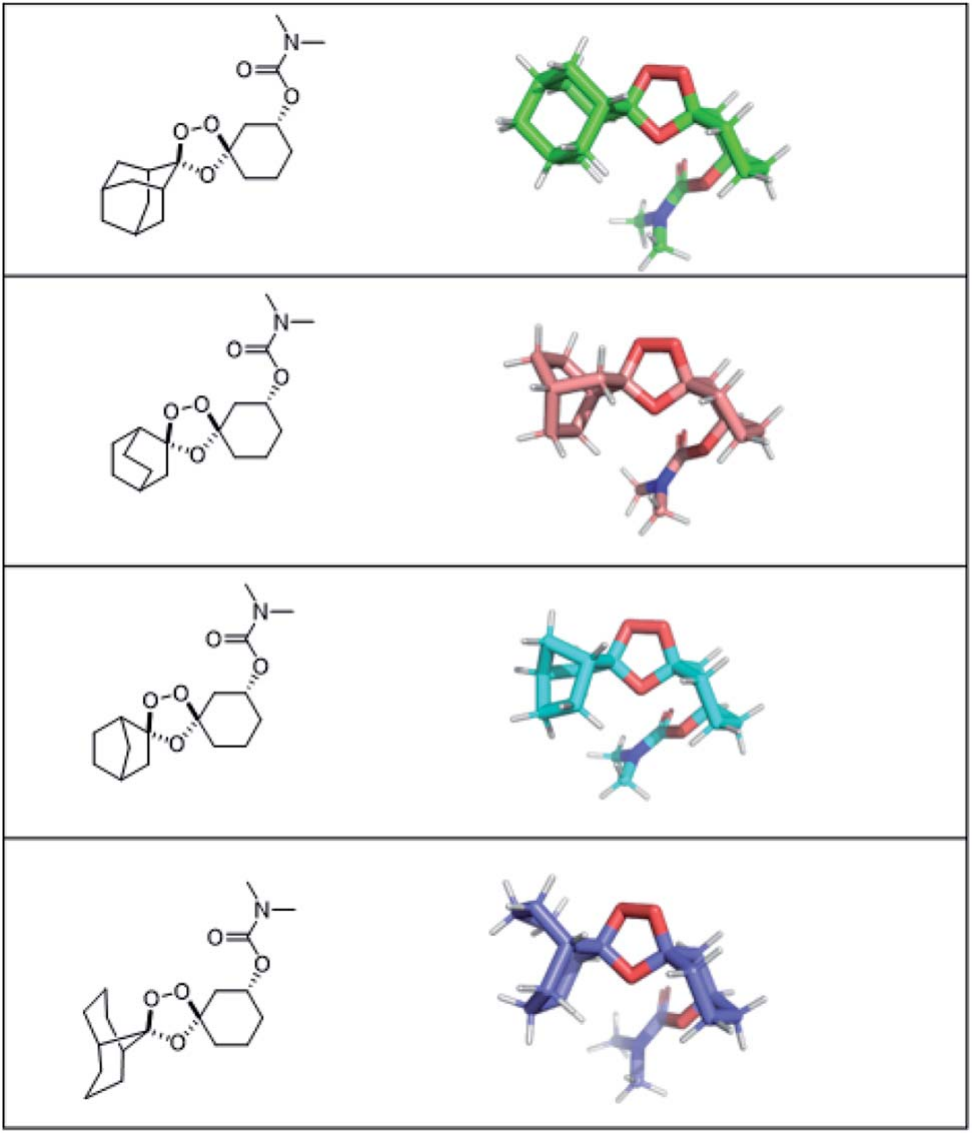


Fig. S1 Representative conformations of putative bridged bicyclic trioxolane adducts, modelled as the *trans-N*,*N*-dimethyl carbamates in the peroxide-exposed, iron(ii)-reactive chair conformation using MarvinSketch (v19.10).

The Royal Society of Chemistry apologises for these errors and any consequent inconvenience to authors and readers.

## Supplementary Material

